# The diet in children with diabetes mellitus (DM)

**DOI:** 10.1186/1824-7288-40-S1-A43

**Published:** 2014-08-11

**Authors:** Dario Iafusco, Angela Zanfardino, Santino Confetto, Francesco Prisco

**Affiliations:** 1 Department of Pediatrics, Second University of Naples, Naples, Italy

## 

A proper nutritional plan is important in the treatment of children and adolescents with diabetes. Its main functions are a) maintaining a good metabolic control, reducing the risk of hyperglycemia and hypoglycemic crisis (induced by prolonged fasting or errors in insulin dose) and b) sustaining the "*long term health*", allowing a good general state of health, normal growth and pubertal development and reducing the risk of micro and macro vascular complications.

The common opinion is that the general characteristics of a diet for a child with diabetes do not differ from the recommended intakes for the general pediatric population (Italian term: LARN), However until a few years ago the adaptation to the recommendations was not entirely possible being necessary to modulate the intake of carbohydrates with the pharmacodynamics of the various types of insulins. The introduction of new insulins such as fast, slow analogs and, at the same time, the increased use of subcutaneous insulin infusion pumps have allowed a better adaptation of the dietary habits of the different ages of life not underestimating that the approach to food by children and adolescents contains emotional meanings (*premium, punishment, gratification, a sense of homologation with peers, etc..*) that may affect the compliance to the therapy. In the basal-bolus insulin regimen it can be very useful to apply the “*rule of carbohydrates*” (adapting the dose of insulin to the amount of carbohydrates in the meal) and the *insulin index* (a parameter which takes into account the effect of amino acids and proteins on postprandial blood glucose levels). The choice of analog rapid or human insulin as boluses in the treatment with multi injection therapy or the possibility to change the bolus type (*square-wave, double-wave,etc.*) in the case of the pump can take in consideration also the timing of absorption of carbohydrates delayed on the basis of the *glycemic index* of food.[1]

However, in line with the global obesity epidemic, a raised weight gain has been described also in adolescents with type 1 diabetes mellitus (T1DM). We have recently shown high prevalence of abdominal adiposity (28,1%) as a sign of metabolic syndrome (MetSy) (9,5%) in adolescents with T1DM [2,3]. On the other hand, due to the prevalence of obesity in children, Type 2 diabetes mellitus (T2DM) in adolescents is becoming an increasingly important public health also in Italy. Diet and exercise are the therapy and prevention of Type 2 diabetes and obesity [4].

## References

[B1] Iafusco D Vanelli M Gugliotta M Iovane B Chiari G Prisco F Prevalence of eating disorders in young patients with type 1 diabetes from two different Italian cities Diabetes Care 2004 27 2278 10.2337/diacare.27.9.2278 15333502

[B2] Valerio G Iafusco D Zucchini S Maffeis C Study-Group on Diabetes of Italian Society of Pediatric Endocrinology and Diabetology (ISPED) Abdominal adiposity and cardiovascular risk factors in adolescents with type 1 diabetes Diabetes Res Clin Pract 2012 97 99 104 10.1016/j.diabres.2012.01.022 22336634

[B3] Valerio G Maffeis C Zucchini S Lombardo F Toni S Rabbone I Federico G Scaramuzza A Franzese A Cherubini V Zedda MA Calcaterra V Lera R Cardinale G Bruzzese M Iughetti L Gallo F De Donno V De Berardinis F Iafusco D Geographic variation in the frequency of abdominal adiposity and metabolic syndrome in Italian adolescents with type 1 diabetes Acta Diabetol 2014 51 163 165 10.1007/s00592-013-0494-6 23807611

[B4] Fainardi V Scarabello C Cangelosi A Fanciullo L Mastrorilli C Giannini C Mohn A Iafusco D La Loggia A Lombardo F Toni S Valerio G Franzese A Prisco F Chiarelli F Vanelli M Physical activity and sedentary lifestyle in children with type 1 diabetes: a multicentre Italian study Acta Biomed 2011 82 124 131 22480067

